# Leaf water potential of coffee estimated by landsat-8 images

**DOI:** 10.1371/journal.pone.0230013

**Published:** 2020-03-18

**Authors:** Daniel Andrade Maciel, Vânia Aparecida Silva, Helena Maria Ramos Alves, Margarete Marin Lordelo Volpato, João Paulo Rodrigues Alves de Barbosa, Vanessa Cristina Oliveira de Souza, Meline Oliveira Santos, Helbert Rezende de Oliveira Silveira, Mayara Fontes Dantas, Ana Flávia de Freitas, Gladyston Rodrigues Carvalho, Jacqueline Oliveira dos Santos

**Affiliations:** 1 Pós-Graduação/Sensoriamento Remoto, Instituto Nacional de Pesquisas Espaciais, São José dos Campos, SP, Brasil; 2 Empresa de Pesquisa Agropecuária de Minas Gerais, Lavras, MG, Brazil; 3 Empresa Brasileira de Pesquisa Agropecuária, Brasília, DF, Brasil; 4 Dept. de Biologia, Universidade Federal Lavras, Lavras, MG, Brasil; 5 Instituto de Matemática e Computação, Universidade Federal de Itajubá, Itajubá, MG, Brasil; CSIRO, AUSTRALIA

## Abstract

Traditionally, water conditions of coffee areas are monitored by measuring the leaf water potential (Ψ_W_) throughout a pressure pump. However, there is a demand for the development of technologies that can estimate large areas or regions. In this context, the objective of this study was to estimate the Ψ_W_ by surface reflectance values and vegetation indices obtained from the Landsat-8/OLI sensor in Minas Gerais—Brazil Several algorithms using OLI bands and vegetation indexes were evaluated and from the correlation analysis, a quadratic algorithm that uses the Normalized Difference Vegetation Index (NDVI) performed better, with a correlation coefficient (R^2^) of 0.82. Leave-One-Out Cross-Validation (LOOCV) was performed to validate the models and the best results were for NDVI quadratic algorithm, presenting a Mean Absolute Percentage Error (MAPE) of 27.09% and an R^2^ of 0.85. Subsequently, the NDVI quadratic algorithm was applied to Landsat-8 images, aiming to spatialize the Ψ_W_ estimated in a representative area of regional coffee planting between September 2014 to July 2015. From the proposed algorithm, it was possible to estimate Ψ_W_ from Landsat-8/OLI imagery, contributing to drought monitoring in the coffee area leading to cost reduction to the producers.

## Introduction

In Brazil, coffee production has great economic and social importance, generating employment, and increasing the population’s income. However, such production is threatened by extreme weather events, such as prolonged droughts and frost. Therefore, coffee plantations need to be constantly monitored in order to establish adequate management practices to minimize production losses. Traditionally, water conditions of coffee areas are monitored by measuring the leaf water potential (ΨW) through a pressure pump. However, measurement is time-consuming, involves high-cost equipment and maintenance, and is applicable only in small areas.

Monitoring the water conditions of coffee plantations requires the use of technologies that allow the evaluation of large areas or regions. In this context, the use of remote sensing presents as an opportunity to quantify drought stress when there is no in-situ weather station available (i.e., for time-series creation) [1,2]. In the past years, the new generation of free medium-resolution satellite imagery such as the Landsat-8/OLI and Sentinel-2/MSI presents suitable information for drought monitoring in agricultural lands [3,4]. Moreover, efforts such as the Harmonized Landsat and Sentinel-2 (HLS) to combine both satellites in a virtual constellation provide a seamless reflectance dataset with a reduced temporal resolution hence offering a high potential for crop monitoring [5].

Recently, Ramoelo et al. [6] proposed modeling techniques to estimate the Ψw of crops using spectral data obtained by remote sensing using the RapidEye sensors. Furthermore, Chemura et al. [7] evaluated a model to estimate plant water content (PWC) in *Coffea arabica* based on field spectrometry. There are also vegetation indices that correlate well with biophysical vegetation parameters and are widely used in estimating biomass, changes in crop development, and are indicative of biotic and abiotic stress [7,8]. For example, the Normalized Difference Vegetation Index (NDVI) is one of the most commonly used vegetation indices in ecological studies as it provides a general measure of vegetation state [9,10]. As these biophysical parameters are related to climate variability [11], NDVI could be used as a surrogate measure of its variability [10,12]. Therefore, several works attempted to explore the relationship between NDVI and other vegetation indices (such as NDWI) with leaf water potential and water stress in different crop cultures. Pu et al. [13] evaluated oak leaves with different water concentrations and observed increased reflectance at wavelengths from 400 to 700 nm and decreased from 750 nm when submitted to water stress. Ramoelo et al. [6] found moderate values for the Pearson correlation between NDVI and Ψw, in dry seasons, using RapidEye images in South Africa, for different species of trees and pasture. As NDVI is sensitive to the presence of chlorophylls and other plant pigments that are responsible for the absorption of red band radiation [14], lower NDVI values under water deficit conditions indicate a decrease in chlorophyll concentration in leaves. Despite NDVI, another commonly used vegetation index for drought monitoring is the Normalized Difference Water Index (NDWI) [1,6,15].

Considering that Ψw is a precise parameter for measuring the water condition of the plant and that the spectral data obtained by remote sensing allows extensive area monitoring, models that establish a relationship between leaf water potential and remote sensing vegetation data that can be used as a monitoring technology of the water conditions of coffee plantations. Therefore, the objective of this study was to propose algorithms to estimate Ψw of coffee areas in Minas Gerais (Brazil) from remote sensing data. To address this objective, the following procedures were performed: i) *in-situ* measurements of Ψw were carried out between 2013–2017 over two cities in Minas Gerais state; ii) Landsat-8/OLI surface reflectance and vegetation indices were correlated with *in-situ* Ψw; iii) Leave-One-Out-Cross-Validation (LOOCV) were used to obtain the performance and applicability of the algorithms; iv) Best algorithm was applied to Landsat-8/OLI imageries for spatialization.

## Materials and methods

### Study area

**Fig 1:**
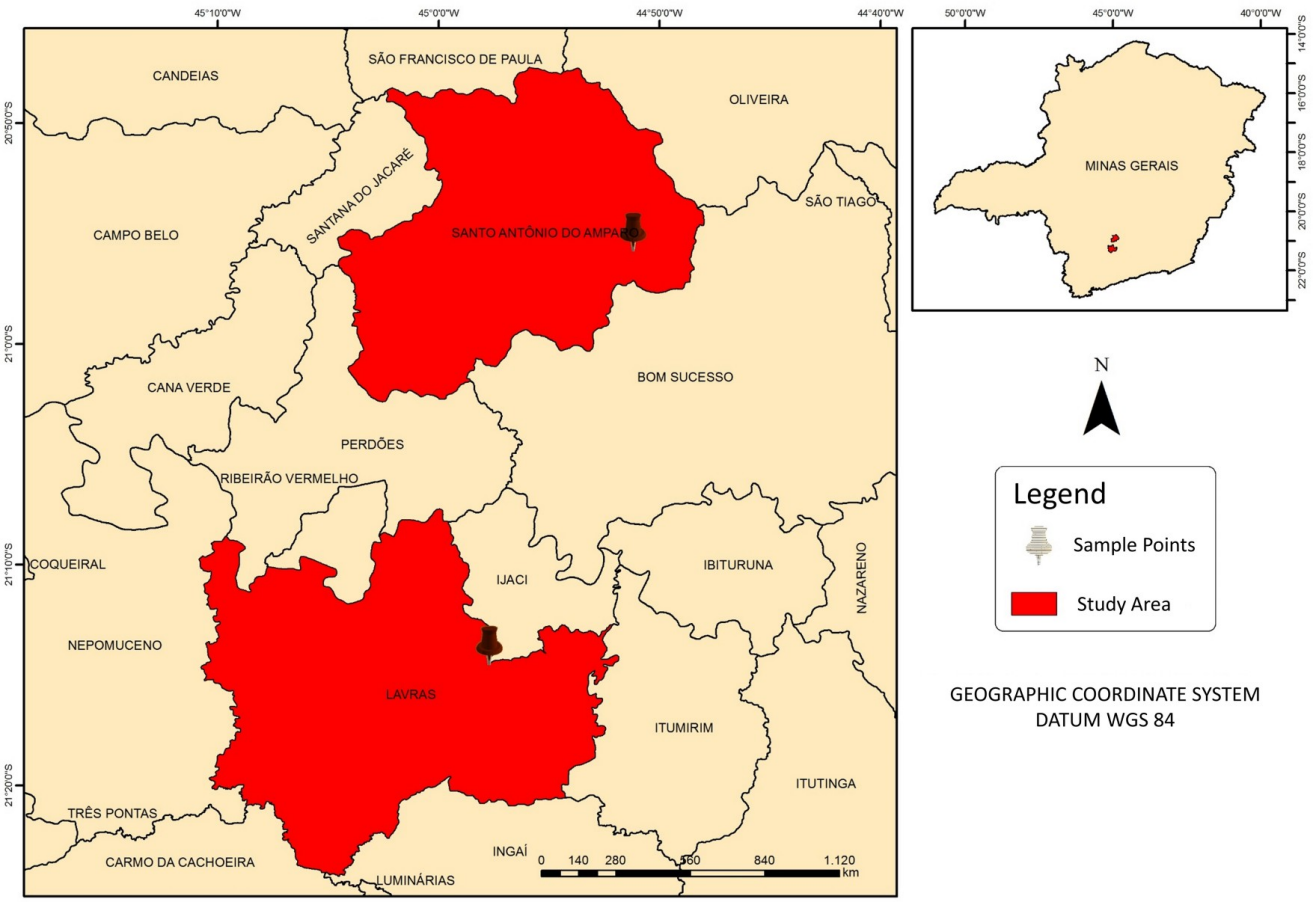
Study area.

The study was conducted in experimental *Coffea arabica* variety Catuaí (spacing of 3,40 x 0,65 m) areas located in the municipalities of Santo Antônio do Amparo and Lavras (Fig 1). Both cities are located in the south region of Minas Gerais, with an average altitude of approximately 950 m. According to the Köppen-Geiser climate classification, the region has a Cwa climate, humid subtropical, with hot and humid summers and cold and dry winters, with an annual average air temperature of 19.4 °C and average annual total rainfall of 1530 mm [16].

Additionally, Ψw (in MPa) was determined following Scholander et al. [17], using a Scholander pressure chamber (1000 PMS Instruments Plant Moisture). All measurements were made in fully expanded leaves of the 3rd or 4th pair from the tip of an actively growing branch (plagiotropic branch). In order to avoid any inhibitory effects of light or temperature on the leaf water potential, the measurements were conducted before dawn (between 04:30 and 05:30), at a mean temperature of 18 °C. Moreover, for the matter of this study, coffee plants were sampled in a 44.2 m^2^ area at each 10 m. Ψw was evaluated for 17 dates, using the mean values of four replicates for the satellite images comparison.

Remote sensing data were obtained from Landsat-8 satellite imagery, Operational Land Imager (OLI). The Landsat-8 satellite was selected due to its spatial resolution and data availability close to the field campaigns. Launched in 2013, OLI sensor provides imagery with 30 m spatial resolution in the visible to shortwave infrared wavelengths (See Table 1), with a revisit time of 16 days [18]. This sensor shows similar characteristics when compared to other sensors from the Landsat program (i.e., Landsat-5 and Landsat-7) with more advanced radiometric and geometric quality.

**Table 1:** Landsat-8/OLI configuration for each spectral band (Barsi et al., 2014).

OLI Bands	Spectral Interval (nm)	Signal-To-Noise Ratio
**B1**	435–451	238
**B2**	452–512	364
**B3**	533–590	302
**B4**	636–673	227
**B5**	851–879	204
**B6**	1566–1651	265
**B7**	2107–2294	334

The images were obtained free of charge via the United States Geological Survey (https://earthexplorer.usgs.gov/) for the path-row 218–75 in surface reflectance (Landsat 8 Surface Reflectance product–L8SR) [19]. L8SR uses an internal algorithm to provide the user with a product with atmospheric correction. The correction used in L8SR is based on the 6SV (Second Simulation of the Satellite Signal in the Solar Spectrum–Vector Version) [19,20] and several authors demonstrated the accuracy of such correction for different targets worldwide, such as vegetation [21,22] and water [23].

Images from the years 2014, 2015, 2016, and 2017 were selected for dates close to the field Ψw collection dates. The spectral bands used are in Table 2. To obtain the surface reflectance values, a pixel was selected in each of the field experiments: (i) Lavras with 21^o^13’40” S; 44^o^57’44” W; altitude 963 m and (ii) Santo Antônio do Amparo: 20^o^54’57” S; 44 ^o^51’13” W; altitude 1090 m. The band values 2, 3, 4, 5 and 6 were extracted in coordinates abovementioned. With the reflectance values, vegetation indices NDVI [24] and NDWI [25] were calculated using Eqs 1 and 2 for NDVI and NDWI, respectively.

$$\mathrm{NDVI}=\frac{R_{850}‐R_{640}}{R_{850}+R_{640}}$$ (1)

$$\mathrm{NDWI}=\frac{R_{850}‐R_{1600}}{R_{850}+R_{1600}}$$ (2)

**Table 2:** Regression models and coefficient of determination (R^2^). Where B represents the satellite’s spectral bands.

Model Name	Models	Pearson r	R^2^
B2_Lin_	Ψw = 0.1266–33.1014 (B2)	-0.85	0.71
B3_Lin_	Ψw = 0.5308–24.4544 (B3)	-0.61	0.33
B4_Lin_	Ψw = 0.4577–24.9085 (B4)	-0.84	0.68
B5_Lin_	Ψw = -2.038 + 3.891 (B5)	0.57	0.28
B6_Lin_	Ψw = 1.473–9.955 (B6)	-0.56	0.27
NDVI_Lin_	Ψw = -4.329 + 4.806 (NDVI)	0.91	0.82
NDWI_Lin_	Ψw = -1.455 + 2.375 (NDWI)	0.74	0.52
B2_Quad_	Ψw = -0.2065–10.9849 (B2) - 267.6433 (B2)^2^	-	0.71
B3_Quad_	Ψw = -3.135 + 107.559 (B3)– 1096.141 (B3)^2^	-	0.48
B4_Quad_	Ψw = -0.0988 + 2.5995 (B4)– 193.7306 (B4)^2^	-	0.69
B5_Quad_	Ψw = -6.825 + 29.639 (B5)– 32.893 (B5)^2^	-	0.36
B6_Quad_	Ψw = -0.8057 + 12.3297 (B6)– 53.3119 (B6)^2^	-	0.23
NDVI_Quad_	Ψw = -8.712 + 17.325 (NDVI) –8.739 (NDVI)^2^	-	0.89
NDWI_Quad_	Ψw = -1.865 + 5.539 (NDWI)– 4.693 (NDWI)^2^	-	0.52

Where R_850,_ R_640,_ and R_1600_ are the reflectance at bands 5, 3 and 6 of OLI sensor with the subscript referring to the center wavelength of each spectral band. Moreover, precipitation data were obtained through a National Institute of Meteorology (INMET) meteorological station located in Lavras (-21.75° and -45.00°).

Table 2 shows the regression models and their respective determination coefficients for Ψw estimation using the spectral bands and the NDVI plant index. The quadratic models showed higher values for the coefficient of determination. For a study in vineyards in the Mediterranean using a field spectroradiometer, Serrano et al. (2010) obtained R^2^ = 0.57. When performing the multivariate analysis, using the VIF selection, the explanatory variables that best fit the multivariate model were bands 2 and both vegetation indices (NDWI and NDVI), with VIF values lower than 5. Therefore, the proposed multivariate model was Ψw = -2.4877–13.753(B2) + 0.26402*NDVI + 0.4254*NDWI, which presented R^2^ = 0.83 (*p* ≤ 0.05; n = 17).

### Statistical analysis and algorithm validation

The statistical relationships between Ψw and remote sensing data were obtained by the coefficient of determination (R^2^) analysis and linear, quadratic, and multivariate models—with variable selected using the Variance Inflation Method (VIF) from R Package [26]. VIF is a widely used tool to measure the degree of multicollinearity between two or more predictor variables [27]. To validate the models, Leave One Out Cross-Validation (LOOCV) technique was applied to all models [28]. LOOCV is a commonly used statistical method for small sample sizes that allow whole samples to be used in training and validation procedures [29,30]. At each step, n-1 samples were used to train the model and another one is used for validation. This process is repeatedly executed until all sample pairs were validated (n = 18 in this work). For each model, Mean Absolute Percentage Error (MAPE) (Eq 3), Root Mean Squared Error (RMSE) (Eq 4), determination coefficient (R^2^) and Pearson r coefficient were calculated.

$$MAPE=100*\bar{\sum_{i=1}^{n}\frac{\left(y_{i}-x_{i}\right)}{xi}}$$ (3)

$$RMSE=\sqrt{\bar{{\left(\sum_{i=1}^{n}(x_{i}-y_{i}\right)}^{2}}}$$ (4)

Where x_i_ is the field measured Ψw values, and y_i_ is the satellite estimated Ψw values for each station after the LOOCV. Therefore, after the algorithm validation, the one with the best results was applied to Landsat-8 imagery using a geographic information system (GIS) and Ψw was therefore calculated. To illustrate the spatialization of estimated Ψw values, a pilot area of approximately 13 km^2^ was selected in Santo Antônio do Amparo, from September 2014 to July 2015.

## Results

### Variability of remote sensing, PH, and precipitation for the study area

**Fig 2:**
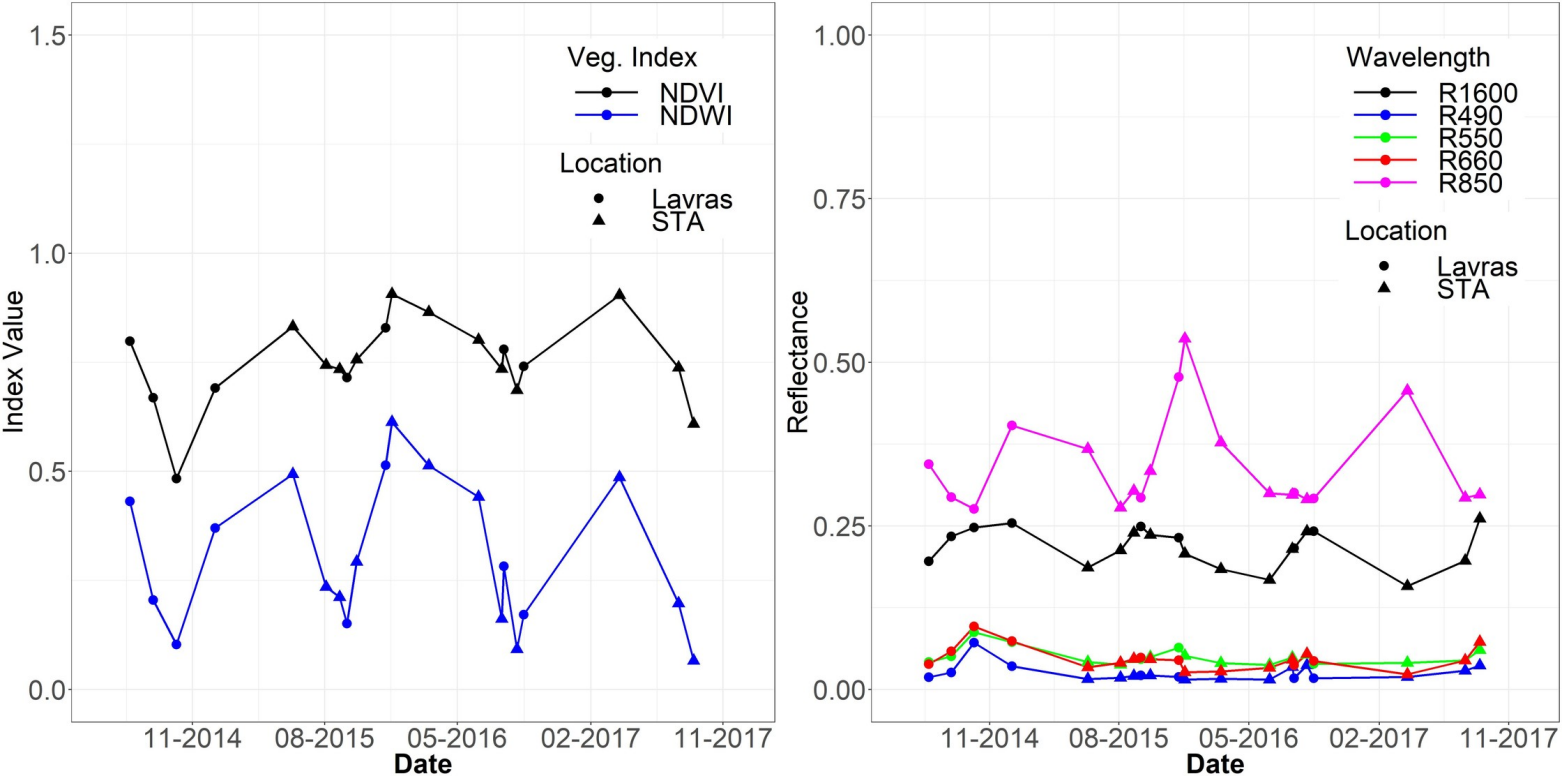
Temporal variability of vegetation indices **(A)** and surface reflectance **(B)** for Lavras and Santo Antônio make Amparo points.

Fig 2 shows the time-series of Landsat-8 reflectance values for both sites of Lavras and Santo Antônio do Amparo for NDVI and NDWI (Fig 2A), and bands 2, 3, 4, 5 and 6 (Fig 2B) for the dates of field surveys. Note that dates for Lavras and STA are referring to dates were Ψw was measured in each site. In the dates corresponding to the drought period in the region (August and September), the reflectance values in bands 2, 3, 4, and 6 increase, and the inverse occurs for band 5 and vegetation indices NDVI and NDWI. It is also important to note the variability of the intensity of NDVI values. For the drought season of 2014 (September 29), NDVI presented a value of 0.48, the lowest value in the analyzed time-series.

**Fig 3:**
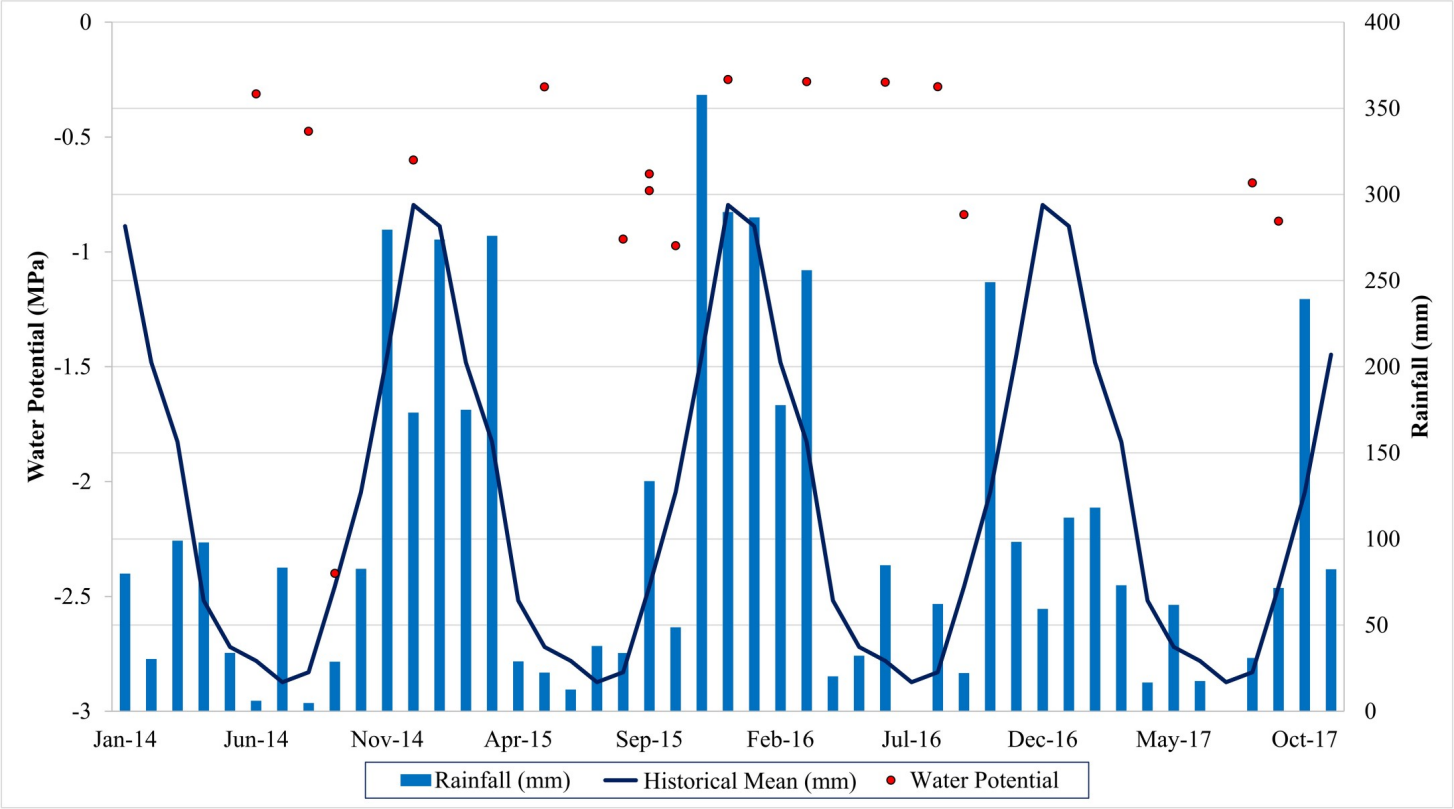
Variability of Ψw (MPa) values, total rainfall (mm), and average mean rainfall (mm), according to the meteorological station (Lavras, MG), from 2014 to 2017.

Fig 3 shows the monthly rainfall that occurred in the studied period, as well as the average normal rainfall for the region and the mean values of Ψw, measured in the field. The variation of Ψw values was from 0 up to -1 MPa, except for September 2014, when the value reached -2.4 MPa. Ψw values follow the observed for the vegetation indices and reflectances (Fig 2A and 2B), as the minimum Ψw was for the same date of minimum NDVI value. On the other dates, Ψw follows the tendency of the spectral response, being high in the rainy period and low at the end of the dry season.

Concerning the precipitation levels, the precipitation in 2014 was much lower than expected, according to the historical mean. The water potential is a crucial water relation parameter that describes the energy state of water; low Ψw is associated with the extent of plant dehydration [31]. Therefore, according to the values of Ψw, the region has favorable climatic conditions to maintain coffee hydration, but the occurrence of low rainfall in 2014 resulted in a moderate water deficit to the plants. Variations in leaf water status may cause alterations in photosynthetic pigment concentrations and photosynthetic activity, in turn, leading to changes in spectral reflectance properties [32].

### Leaf water potential algorithms

The best Pearson correlations were between the values of Ψw and the spectral bands of the visible B2 (R = -0.85), B3 (R = -0.61), and B4 (R = -0.84) (Table 2). There was a strong negative correlation indicating that for smaller Ψw values, a higher reflectance occurs in these bands. The results obtained for the bands of blue and red (bands 2 and 4, respectively), characterize a higher reflectance in the absorption bands of chlorophyll, indicating a smaller photosynthetically active area. Drought stress stimulates earlier leaf senescence, particularly in physiologically older leaves. Besides, this drought stress can decrease the net photosynthetic rate per unit leaf area. These decreases are strongly associated with stomatal factors, as coffee stomata are quite sensitive to both soil water availability and evaporative air demand [31].

### LOOCV results and spatialization

Then, the models were applied to the LOOCV technique in order to validate the empirically developed algorithms. The results of the LOOCV were presented in Table 3. For most of the algorithms, the results were not accurate, with R^2^ values lower than 0.6 and MAPE and RMSE values high, indicating errors of up to 50% in the Ψw estimate (MAPE values higher than 50% were not shown in Table 3 for brevity).

**Table 3:** Statistical results obtained through the LOOCV.

Model Name	MAPE (%)	R^2^	Pearson r	RMSE (Mpa)
MV*	48.97	0.18	0.48	0.48
B4_Lin_	44.63	0.39	0.66	0.39
NDVI_Lin_	45.23	0.67	0.83	0.29
NDWI_Lin_	37.18	0.34	0.62	0.41
B4_Quad_	49.79	0.05	-0.14	0.65
NDVI_Quad_	**27.09**	**0.85**	**0.93**	**0.21**
NDWI_Quad_	31.33	0.24	0.54	0.46

Values in bold indicate the best results for each statistical metric.

*Multivariate Model

**Fig 4:**
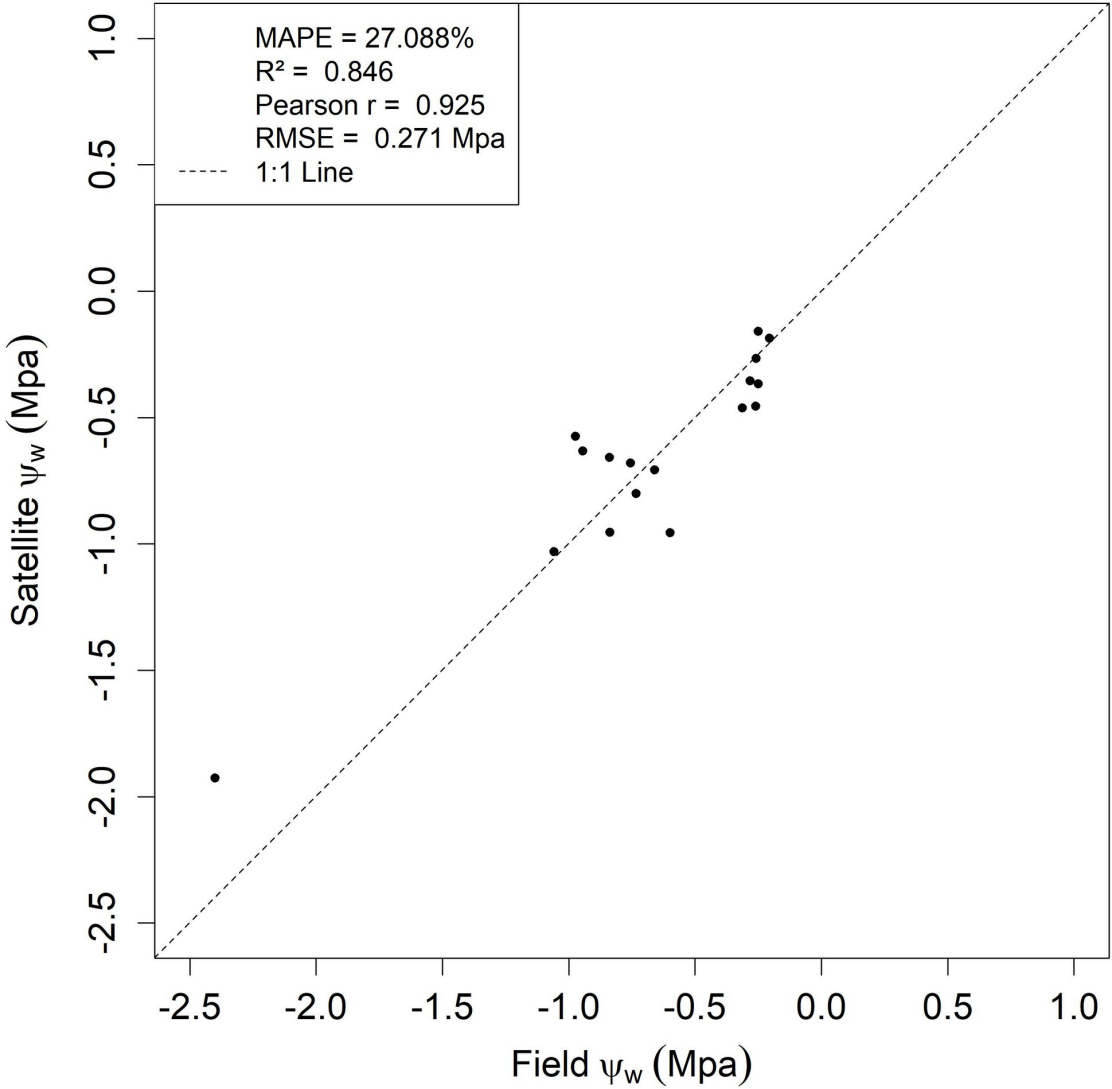
LOOCV results for the NDVI_Quad_ algorithm. The upper left box refers to MAPE, R^2^, Pearson r, and RMSE for the validation using LOOCV.

The best result obtained for the LOOCV was for the Quadratic NDVI algorithm (NDVI_Quad_), with errors lower than 30%. Moreover, a good agreement between the field-measured Ψw and the predicted Ψw by OLI sensor (R^2^ = 0.84, Pearson r = 0.92) was observed. These results are in agreement with the exploratory analysis (See Table 3), in which NDVI_Quad_ was the best regression to estimate Ψw. Fig 4 shows the results of the LOOCV using the NDVI_Quad_ algorithm, with the satellite predicted Ψw and the values obtained in the field.

**Fig 5:**
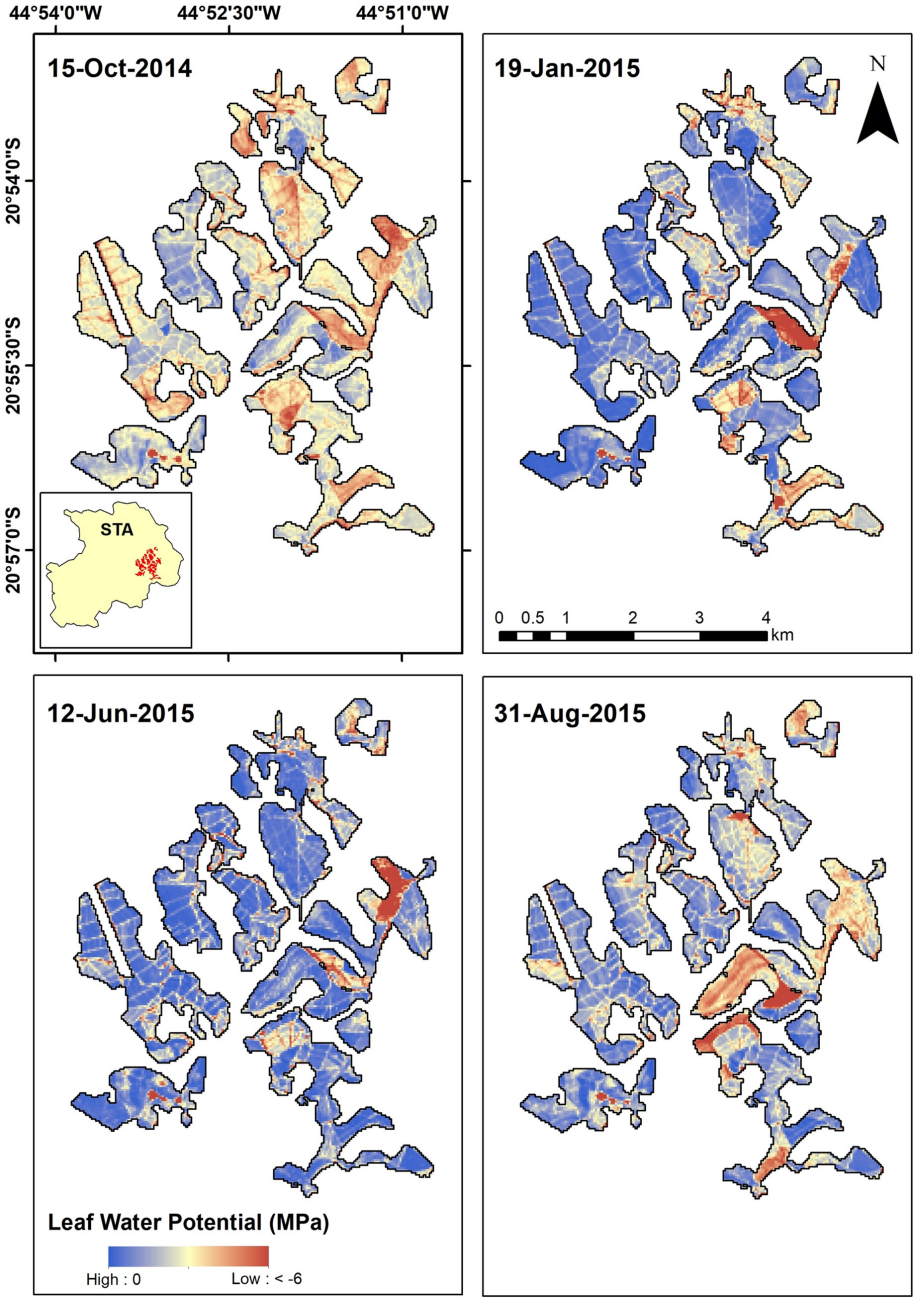
Ψw estimated (MPa) between September 2014 and July 2015, in an area representative of the study region, in Santo Antônio do Amparo.

Thus, with the best results for the NDVI_Quad_ model, it was inserted into the geographic information system (GIS) and the values of Ψw were estimated from Landsat-8 OLI images between September 2014 and July 2015, corresponding to a dry and rainy period, respectively. Fig 5 shows the map of estimated Ψw values in an area representative of the study region. It was estimated that, during the dry season (September/2014), the mean Ψw value was -0.91 ± 0.35 MPa. For January 2015, as precipitation increases, the mean Ψw was -0.70 ± 0.29 MPa. The increase in Ψw values was also observed until June 2015, with mean estimated Ψw of -0.50 ± 0.25 MPa. With the end of the rainy season, mean Ψw starts to decrease again (mean values of -0.61 ± 0.25 MPa). Furthermore, as orbital remote sensing provides a synoptic view of land areas, the spatial variability of Ψw can be beneficial for planning coffee management.

## Discussions

The currently widely used method to assess plant drought and water status is the pressure chamber. However, this method has the limitations of being destructive, point-based, and user-dependent, which restricts large areas of monitoring. In this work, we provided a Landsat-8/OLI-based Ψw algorithm using NDVI to predict Ψw with reliable accuracy (MAPE < 30%, R^2^ > 0.85, RMSE < 0.21 Mpa). Therefore, it was possible to apply the algorithm and obtain a synoptic view of an experiment area, which could contribute to cost reduction in coffee water status management.

The use of NDVI as an indicator of drought vegetation stress and soil moisture was already reported by several authors [2,9,10,12] as NDVI provides a general measurement of vegetation state and health and has been used for accessing drought status since the 1970s when this index was proposed by Rouse [33]. However, there is also a difficulty in monitoring water stress using vegetation indices as this response is observed when notable damage to the culture has already occurred [10].

When the plant is submitted to a water stress condition, NDVI values tend to decrease as water conditions alter the biophysical conditions in the leaves. Despite near-infrared and red bands not being directly correlated with the water content, they are linked to chlorophyll and other biophysical parameters such as aboveground net primary production [34], green leaf biomass and leaf photosynthetic activity [35] and these variables are linked to water stress [36]. Gu et al. [37] found a high correlation (r = 0.73) between fractional water index (FWI) and both NDVI and NDWI for sites surrounded by relatively homogeneous vegetation with silt loam soils at Oklahoma, USA. Furthermore, Mbatha and Xulu (2018) also demonstrate the applicability of NDVI to monitor the impact of intense drought in South Africa due to El Niño effects. The results obtained for the quadratic NDVI model in this work were better than those obtained by Ramoelo et al. (2015) and better than those obtained by Rallo et al. (2014), with R^2^ = 0.36 and RMSE of 0.44 MPa, and Cotrozzi et al. (2017), with R^2^ = 0.65 and RMSE of 0.51 MPa, using a field spectroradiometer.

## Conclusions

In this work, we provided an empirical algorithm for estimate Leaf Water Potential (Ψw) using Landsat-8 surface reflectance and vegetation indices data for Coffee Arabica areas in Minas Gerais state, Brazil. From the validation, a quadratic NDVI algorithm presented the best result for Ψw estimative, with Mean Absolute Percentage Error (MAPE) of 27.09% and an R^2^ of 0,85, being, therefore, an option to estimate Ψw of coffee areas from the surface reflectance obtained from the Landsat-8 satellite OLI sensor. The spatialization of the estimated Ψw values in the region is a technology that can enable the satellite monitoring of water conditions of coffee plants to establish appropriate practices, such as irrigation economics, pest and disease control, and fertilization management, allowing environmental and economic sustainability of coffee plantations in the largest coffee region of Brazil.

## Supporting information

S1 Data (XLSX)
